# Editorial: The role of cell metabolism in development, drug resistance, and survival assessment in cancer

**DOI:** 10.3389/fmolb.2025.1685924

**Published:** 2025-10-07

**Authors:** Kishor Pant, Ashok Kumar

**Affiliations:** ^1^ The Hormel Institute (University of Minnesota), Austin, MN, United States; ^2^ Department of Biochemistry, All India Institute of Medical Sciences Bhopal, Bhopal, India

**Keywords:** cellular metabolism, drug resistance, cancer biology, aerobic glycolysis, warburg effect, tumor mi croenvironment

## Overview

Cellular metabolism is a crucial and increasingly established area of cancer research that significantly influences disease progression and supports tumor proliferation. Genetic alterations are usually crucial to cancer biology; however, it is now evident that tumor cells undergo significant metabolic reprogramming to promote proliferation, adapt to stress, and increase metastasis (Hanahan and Weinberg, 2011). Conditions such as Wilson’s Disease underscore the importance of metabolic equilibrium. Tumorigenesis entails intricate alterations in the tricarboxylic acid (TCA) cycle, oxidative phosphorylation, amino acid metabolism, and lipid metabolism (Ward and Thompson, 2012; Vander Heiden et al., 2009). Nonetheless, the mechanisms by which these processes directly promote proliferation, metastasis, and resistance to therapy remain poorly elucidated. This Research Topic, “The Role of Cell Metabolism in Development, Drug Resistance, and Survival Assessment in Cancer,” includes research articles and reviews exploring metabolic pathways that affect carcinogenesis, progression, drug resistance, and prognosis. The studies included critically address the function of metabolic pathways in treatment resistance and the potential of metabolomics for biomarker identification. Together, these contributions enhance our understanding of metabolism’s influence on cancer and highlight novel treatment possibilities.

## Metabolic reprogramming: promoting tumor growth

Cancer cells are metabolic opportunists that modulate glucose, lipid, and amino acid pathways beyond the traditional Warburg effect. The articles in this special issue illustrate how metabolic reprogramming drives carcinogenesis, metastasis, and chemoresistance.

For example, Niu et al. showed that fucoxanthin, a marine carotenoid, reduces acute myeloid leukemia cell proliferation by inhibiting AKT signaling and diminishing GLUT1-mediated glucose absorption (Front Pharmacol 2025). These findings demonstrate how the disruption of metabolic nodes reveals therapeutic vulnerabilities.

Wang et al. investigated methylglyoxal (MG), a byproduct of glycolysis with dual functions in cancer. Depending on the concentration and context, MG can either promote or inhibit tumor development, indicating its therapeutic potential in controlling cell growth.

A number of studies discovered prognostic metabolic markers. Wang et al. identified a reprogramming-based signature in small cell lung cancer (SCLC), connecting MOCS2 to chemotherapy sensitivity and immunological interactions (Front Mol Biosci 2025). Jiang et al. identified a metabolic profile based on MS4A7 as a predictive factor in lung cancer. Furuhashi et al. emphasized volatile organic compounds (VOCs) as non-invasive metabolic indicators with potential for diagnosis and monitoring of illness.

## Metabolic mechanisms and chemoresistance

Drug resistance remains a significant obstacle to cancer treatment, and is sometimes associated with metabolic adaptability (Vasan, 2019). The articles below underscore the necessity of identifying metabolic vulnerabilities to overcome resistance.

Niu et al. revealed that inhibiting AKT-mediated glucose metabolism may resensitize resistant cancers. Ma et al. assessed intra-tumor heterogeneity (ITH) in prostate adenocarcinoma (PRAD), demonstrating that metabolic vulnerability promotes treatment resistance and emphasizing MYLK2 as a potential therapeutic target. Wang et al. examined MG’s dual function in resistance, showing that its pro-tumorigenic effects are more significant in resistant cancers (Front Mol Biosci 2025). Taken together, these studies underscore that metabolic variability and plasticity contribute to therapeutic failure, and efforts to impede these adaptations are essential for sustained treatment efficacy.

## Clinical metabolomics, biomarkers, and personalized therapy

Clinical metabolomics, which analyzes metabolites as immediate markers of tumor physiology, shows great promise in diagnosing, prognosing, and personalizing cancer therapy.

Wang et al. established that metabolic fingerprints can predict the prognosis of small cell lung cancer (SCLC) and characterize immunological landscapes, suggesting that resistant and susceptible tumors show unique metabolic states. Jiang et al. confirmed that MS4A7-based gene expression patterns serve as prognostic markers in lung cancer. These studies underscore the prognostic significance of metabolomics for patient outcomes.

The research by Furuhashi et al. on volatile organic compounds (VOCs) illustrated a viable non-invasive biomarker platform. VOCs, which are identifiable in patient biofluids, could facilitate early diagnosis, progression tracking, and real-time evaluation of therapy.

The study conducted by Ma et al. further substantiated the combination of metabolomic data with genetic profiles to formulate tailored therapies aimed at addressing specific metabolic vulnerabilities. These tactics seek to optimize efficacy and reduce toxicity.

## Conclusions and prospective direction

The research included in this Research Topic emphasizes cellular metabolism as a fundamental catalyst of cancer formation, progression, and treatment resistance, rather than merely an outcome of malignancy. These studies collectively explain how metabolic reprogramming regulates tumor development, contributes to heterogeneity, and affects treatment results, while also emphasizing the potential of clinical metabolomics and non-invasive biomarkers for prognosis and customized therapy. In the near future, the integration of metabolomic data with genetic and proteomic information will be essential for identifying new metabolic hubs, enhancing treatment approaches, and addressing resistance. Improving our comprehension of the interactions among metabolism, tumor biology, and the immune response will bring us closer to novel therapies that could improve survival and quality of life for patients.
